# Heterogeneity in the Epigenetic Landscape of Murine Testis-Specific Histone Variants TH2A and TH2B Sharing the Same Bi-Directional Promoter

**DOI:** 10.3389/fcell.2021.755751

**Published:** 2021-12-06

**Authors:** Isha Singh, Priyanka Parte

**Affiliations:** Department of Gamete Immunobiology, ICMR-National Institute for Research in Reproductive Health, Mumbai, India

**Keywords:** TH2a, TH2b, H3K4me2/3, NUMTs, epigenetic inheritance, spermatogenesis, embryogenesis

## Abstract

Testis-specific histone variants are crucial to promote open chromatin structure to enable nucleosome disassembly in the final stages of spermiogenesis. However, even after histone replacement, mature sperm retain a proportion of these variants, the function of which is unknown. The present study aimed to understand the functional relevance of the retained H2B and H2A variants, TH2B and TH2A. While no literature is available on the phenotype of TH2A knockouts, TH2B/TH2A double knockout male mice are reported to be infertile. In this study, ChIP-seq analysis was done for TH2B and TH2A to understand the epigenomics of the retained TH2B and TH2A, using murine caudal sperm. Distribution across genomic partitions revealed ∼35% of the TH2B peaks within ±5 kb of TSS whereas TH2A peaks distribution was sparse at TSS. Gene Ontology revealed embryo development as the most significant term associated with TH2B. Also, based on genomic regions, TH2B was observed to be associated with spindle assembly and various meiosis-specific genes, which is an important finding as TH2A/TH2B DKO mice have been reported to have defective cohesin release. A comparison of mouse and human TH2B-linked chromatin revealed 26% overlap between murine and human TH2B-associated genes. This overlap included genes crucial for embryogenesis. Most importantly, heterogeneity in the epigenetic landscape of TH2A and TH2B was seen, which is intriguing as TH2B and TH2A are well reported to be present in the same nucleosomes to promote open chromatin. Additionally, unlike TH2B, TH2A was enriched on the mitochondrial chromosome. TH2A was found to be associated with Nuclear insertion of Mitochondrial DNA sequences (NUMTs) in sperm. A comprehensive analysis of these observations indicates novel functions for the sperm-retained TH2B and TH2A.

## Introduction

Mature sperm and round spermatids harbor the same haploid genome but different epigenomes. Developmental limitations of embryos resulting from somatic cell nuclear transfer (SCNT) and round spermatid injection (ROSI) as compared to sperm borne embryos reveal the importance of the unique sperm chromatin in efficient embryogenesis (Kimura and Yanagimachi, 1995; Kishigami et al., 2004; Teperek et al., 2016). The negative effects of paternally derived transgenerational epigenetic inheritance on fertility and development are linked to disrupted sperm histone methylation, altered sperm RNAs, and retained sperm histone in both mice and men (Gapp et al., 2014; Siklenka et al., 2015; Denomme et al., 2017; Lismer et al., 2020). That embryonically important loci like Homeobox (HOX) cluster escapes epigenetic reprogramming during spermiogenesis underpins the importance of sperm epigenetics (Arpanahi et al., 2009; Hammoud et al., 2009; Erkek et al., 2013; Teperek et al., 2016; Murphy et al., 2018; Yamaguchi et al., 2018; Singh et al., 2021). The loci important for embryonic development and sperm function are marked by nucleosomes containing modified histones like H3K4me3 and H3K27me3. The chromatin states of these respective loci are correlative to their expression in early embryos (Brykczynska et al., 2010; Jung et al., 2017; Oikawa et al., 2020).

Nucleosome, being an octamer, contains two copies each of H2A, H2B, H3, and H4 draped by two turns of duplex DNA (Luger et al., 1997). The portion of histones retained in sperm constitute 4%–10% in men and vary from 1% to 10% in mice (Hammoud et al., 2009; Brykczynska et al., 2010; Jung et al., 2017; Yamaguchi et al., 2018). Apart from the modified histones and evolutionarily conserved H4, the repertoire of retained sperm histones comprise other canonical as well as testis-specific histone variants like H3t, H2A.X, H1T, H1LS1, H2AL2, TH2A, and TH2B (Santenard and Torres-Padilla, 2009; Bao and Bedford, 2016; Wang et al., 2019). Most of these histone variants are indispensable for nucleohistone to nucleoprotamine transition as depicted by their respective knockout mouse models (Patankar and Parte, 2017; Wang et al., 2019).

**FIGURE 1:**
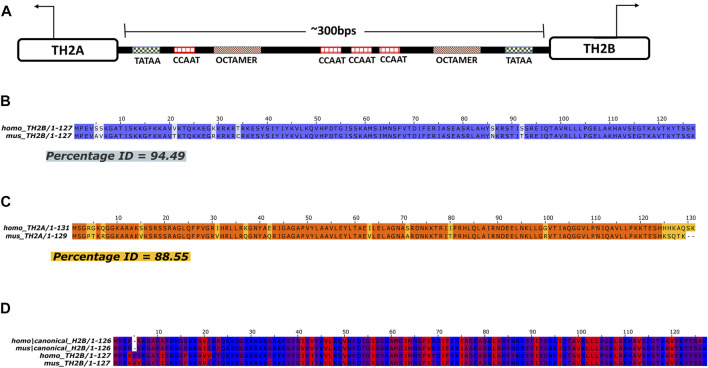
Pairwise Alignment of TH2B and TH2A in Human and Mouse. **(A)** Structure of the TH2A and TH2B gene showing bi-directional promoter (direction of transcription indicated by arrows) with protein binding sites. **(B, C)** Pairwise alignment of protein sequences maps to depict homology between homo (human) and mus (mouse) TH2B and TH2A, respectively. **(D)** Clustal omega Multiple Sequence Alignment (MSA) map for homo and mus H2B and TH2B depicting hydrophobicity of each residue in the protein sequence. Red being hydrophobic and blue being hydrophilic residues.

Germ cell-specific variants of H2B and H2A, namely, TH2B (Hist1h2ba) and TH2A (Hist1h2aa), were first reported in mammalian testis (Shires et al., 1976; Trostle-Weige et al., 1982). Subsequently, significant amount of these histone variants was found to be present in oocyte and zygote, with TH2A/TH2B levels decreasing as embryos differentiate into blastocyst (Shinagawa et al., 2014). Both the histone variants share the same bi-directional promoter (Figure 1A). There is ∼92.4 and 88.5% identity between H2A and TH2A in human and mice, respectively (Supplementary Figure S1A,B), whereas somatic H2B and TH2B are almost 85 and 86% identical in humans and mice (Supplementary Figure S1C,D). TH2B or H2B.1 is the chief histone variant of somatic H2B and almost completely replaces it during spermiogenesis.

Crystal structure analysis comparing nucleosomal core particles (NCP) revealed that TH2A/TH2B containing nucleosomes have decreased hydrogen bonds and fewer DNA-histone contacts as compared to canonical counterparts. This leads to a more open chromatin in TH2A/TH2B nucleosome (Padavattan et al., 2015, 2017). In literature, to date, there are no reports of TH2A single knockout mouse whereas epididymis of a C-terminally modified TH2B mouse model shows absence of spermatozoa rendering the mice sterile. However, TH2B null mice are fertile with compensatory increased levels of somatic H2B and nucleosome destabilizing modifications like lysine crotonylation and arginine methylation (Montellier et al., 2013). Presence of TH2B in chromatin is attributed to nucleosome destabilization, thus laying the foundation for efficient histone to protamine replacement (Govin et al., 2007). Mutant oocytes lacking TH2A/TH2B show decreased blastocyst development as compared to wild-type counterparts in spite of no defects in oogenesis and folliculogenesis in the mutant. Moreover, parthenogenetic development indicated no effect of TH2A/TH2B on maternal genome activation. However, delayed Nanog-GFP expression from the paternal genome in mutant embryos revealed that TH2A/TH2B are indeed involved in paternal genome activation (Shinagawa et al., 2014).

The TH2A/TH2B duo is crucial for spermatogenesis and chromatin structure. Double knockout mouse of TH2A and TH2B show male sterility owing to loss of cohesin release during interkinesis and defects in transition protein 2 (TP2) incorporation and histone replacement (Shinagawa et al., 2015). These lines of evidence suggest that TH2A and TH2B may be working together in regulating the chromatin architecture during spermatogenesis. Montellier and others have used round spermatids for germ cell ChIP-Seq (Montellier et al., 2013). However, there is not much insight into the TH2A/TH2B retained in the mature sperm. It is important to note here that the histone replacement is complete in the caudal sperm and there is no difference in histone retention in sperm from vas deferens and caudal epididymis (Yoshida et al., 2018). The retained histone marks in the sperm are suggestive of the past events that happened during genesis of the sperm as well as the future ones crucial to embryogenesis. The importance of these histones in histone eviction is already established whereas why these are retained is yet to be answered. Genome-wide mapping of sperm TH2A/TH2B will be crucial to understand their functioning and their importance in the mature sperm and subsequent embryo development. To answer these questions, in the present study, mapping of TH2A and TH2B was done in the mouse sperm using chromatin immunoprecipitation sequencing (ChIP-Seq). Our observations indicate that contrary to the existing belief, TH2A and TH2B have relatively fewer overlapping genomic regions. TH2A is majorly found in the intergenic region, whereas apart from the intergenic regions, TH2B is mainly associated with promoter proximal and exonic sequences. It is found to be associated with spindle assembly genes, which is an important finding as germ cells of TH2A/TH2B double knock out mice show cohesin retention. Also, TH2B has been found to be enriched at various spermatogenic and embryologically important loci. Lastly, we have compared genome-wide association of murine TH2B with human TH2B occupancy in mature sperm because of the discrepancies in histone retention in human and mouse sperm. We have tried to find the importance of loci that are commonly retained between the homologs. The detailed results are presented henceforth.

## Materials and Methods

### Experimental Model and Ethics Statement

BALB/c-129-E mice (8–12 weeks old) were used in this study. Mouse husbandry and experimental procedures were carried out as per the guidelines of the National Institute for Research in Reproductive Health Institutional Animal Ethics Committee (NIRRH-IAEC). All animal experiments were approved by the NIRRH-IAEC (project no. 07/18). Mice were housed in groups of four per cage under conditions of 12-h light–dark cycle and provided with water and food *ad libitum*. For experiments, mice were euthanized *via* cervical dislocation.

### Sperm Isolation From the Cauda Epididymis

Mouse sperm were isolated as per the protocol described by Hisano et al. (2013). The caudal region of the epididymis was dissected from adult mice to retrieve mature sperm. The cauda epididymides were briefly rinsed in 0.1 M phosphate buffered saline (PBS) to clear away blood vessels and fats and placed in a culture dish containing PBS, incised 8–10 times, and incubated at 37°C with gentle shaking for 1 h to assist sperm to swim out. The sperm suspension was then filtered through a 40-µm cell strainer, transferred to a tube and washed thrice with PBS by centrifugation at 800 g*30 min at 4°C to eliminate somatic cells, if any. Quantity and purity of sperm was determined using Neubauer chamber. On an average, sperm yield per mouse (i.e., two cauda epididymis) was ∼5–6 million.

### Western Blotting

Western blot analysis of TH2A and TH2B was done to detect their presence in mouse caudal sperm (mature sperm). Whole testicular cell lysates were used as positive control. Towards this, after removing the tunica albuginea, the seminiferous tubules were teased out in PBS and incubated at 37°C for 1 h at 70 rpm. Following this, the testicular cell suspension was filtered through 40-µm Falcon™ Cell Strainers (Fisher scientific) and pelleted by centrifugation at 2,000*g* for 10 min at 4°C. The pellet was washed thrice by centrifugation as above and resuspended in 250 µl of 2D lysis buffer containing 7 M urea, 2 M thiourea, 4% CHAPS, 0.1 M DTT and protease inhibitor cocktail (Roche).

Caudal sperm (∼8 million cells) collected by the sperm swim-up procedure were initially treated with 10 mM dithiothreitol (DTT) in PBS at room temperature for 1 h to release the protamines. The DTT pre-treated sperm were then pelleted by centrifugation at 2,000 *g* for 10 min at 4°C, pellet washed as described above, and resuspended in 150 µl of 2D lysis buffer. Caudal sperm suspension and whole testicular cell lysate were incubated on a rotary shaker overnight at 4°C. The following day, the lysates were subjected to bead beating homogenization in a Fast prep-24 homogenizer (MP Biomedicals). The homogenates were incubated on ice for an hour and then centrifuged at 16,000 *g* for 30 min at 4°C. The supernatants containing the protein lysate were quantified for protein content using the 2-D Quant Kit (Cytiva, 80-6483-56—Sigma-Aldrich).

For Western analysis, 200 µg of caudal sperm for TH2A and 100 µg for TH2B and 50 µg testicular cell lysates were resuspended in Laemmli buffer containing 25% 2-mercaptoethanol, 0.01% bromophenol blue, 50% Glycerol, 20% SDS, and 0.25 M Tris-HCl, pH 6.8. Samples were heated for 5 min at 95°C and subjected to 15% SDS-PAGE at 100 V for 2 h and *trans*-blotted on nitrocellulose membrane at 100 V for 1 h Non-specific binding to the membrane was blocked with 3% NFDM in 0.1 M PBS (blocking buffer) for 1 h at room temperature. Next, these membranes were incubated with primary antibody overnight at 4°C, followed by two washes with PBS containing 0.05% Tween 20 (PBST). The membranes were subsequently incubated with HRP-conjugated secondary antibody for 1 h at RT. The unbound antibodies were eliminated by washing the membrane thrice with 0.05% PBST. Chemiluminescence detection of protein of interest was done using Western Blot Chemiluminescence HRP substrate (Takara-bio), in ChemiDoc imager (Bio-Rad).

Primary antibodies used were rabbit anti-mouse TH2A (a kind gift from Dr. Toshie Shinagawa, RIKEN) at a dilution of 1:250 and rabbit anti mouse TH2B Antibody (Sigma-Aldrich #07–680) at a dilution of 1:5,000. Secondary antibody used was HRP-conjugated swine anti-rabbit IgG (Dako) at a dilution of 1:1,500 in 1% blocking buffer.

### Sperm Chromatin Immunoprecipitation

The protocol described by Hisano et al. (2013) was followed for ChIP in mice sperm with slight modifications (Hisano et al., 2013). Each ChIP experiment was done in two experimental replicates that were pooled before sequencing, for optimization of yield. For each ChIP experiment, 12 million sperm cells isolated from cauda epididymis of adult male mice, were used. A total of 48 million sperm were used for four ChIP reactions, two each for TH2A and TH2B. The 48 million sperm were divided in 24 aliquots of 2 million sperm each. The sperm were washed in 0.1 M PBS and subjected to pretreatment with 50 μM dithiothreitol for 2 h at RT to reduce the disulfide bond between protamines. The sperm were washed in PBS, lysed using 0.5% (vol/vol; final conc.) Nonidet P-40 (Abcam #ab142227) and 1% (wt/vol final conc.) sodium deoxycholate [DOC; Sisco Research Laboratories #96876 (0447,144)]. MNase digestion was performed using 15 U of Micrococcal Nuclease (MNase; New England Biolabs, #M0247S) per 2 million sperm for 5 min at 37°C in a thermomixer.

The MNase digestion was stopped by addition of 0.5 M EDTA. The digestion mixture was centrifuged at 16,000 *g* for 10 min at 4°C to pellet down the denser protamine-bound DNA, and the supernatant containing mononucleosomal chromatin was retrieved. Preclearing of the MNase-digested chromatin was done with blocked protein-A Sepharose beads (GE Healthcare Cat #17-6002-35 Lot no. 10247535) for 1 h. The chromatin (from 12 million sperm in two replicates) was then immunoprecipitated overnight with 6 µg each of either TH2A or TH2B antibody. The following day, blocked beads were added and incubated with end-to-end mixing at 4°C for 4 h, to capture the antibody chromatin complexes.

The antibody–bead complexes were washed by centrifugation (Eppendorf, Germany; Cat# 0030108051) and the chromatin was eluted in two rounds in Tris-EDTA buffer, pH 8.0, containing 10 mM Tris-HCl, 1 mM disodium EDTA, and sodium dodecyl sulfate (1%) by centrifugation at 11,000 *g* for 2 min at RT. The eluted (∼300 µl) immunoprecipitated chromatin DNA was treated with 6 µl of RNaseA (10 mg/ml) at 37°C for 30 min and overnight incubation at 56°C with 6 µl of proteinase K (10 mg/ml) and extracted *via* column-based DNA extraction. Mononucleosomal DNA (one-tenth of the IP reaction) was saved as input. DNA quality and concentration were evaluated using Qubit Fluorometric Quantification (Invitrogen Qubit™ four Fluorometer #Q33226). The entire eluate was mixed with 6× DNA loading dye (final conc. 1×) and electrophoresed on a 5% polyacrylamide gel for 45 min at 100 V with 0.1 M TBE as electrode buffer. The gel was post-stained with ethidium bromide. Mononucleosomal DNA band at ∼150 bp was excised and the mononucleosomal DNA was re-precipitated and subjected to sequencing after pooling the replicates.

### Next-generation ChIP sequencing and preprocessing of sperm ChIP data.

TH2A and TH2B library preparation was done and the quality assessment was performed on automated electrophoresis system using TapeStation Analysis Software A.02.02 (SR1) (Agilent). Sequencing was performed with Illumina Hiseq using 150 bp paired end sequencing. For Input and TH2B ChIP-Seq libraries and for TH2A, ∼30 million and 34 million unique paired end reads were attained, respectively. The following parameters from fastq file were determined using FastQC (www.bioinformatics.babraham.ac.uk/projects/fastqc): 1) Base quality score distribution, 2) Sequence quality score distribution, 3) Average base content per read, 4) GC distribution in the reads, 5) PCR amplification issue, and 6) Checking for overrepresented sequences (Andrews et al., 2015). Based on quality report of fastq, files were trimmed using Trimmomatic (*Ver-0.36*) (Bolger et al., 2014) to retain only high-quality sequences for further analysis. The paired-end reads were aligned to the reference *Mus* Musculus (mm10) release downloaded from Sanger Institute database. Alignment was performed using BWA MEM (*Ver-0.7.12*) (Langmead and Salzberg, 2012). The alignment percentages for TH2A, TH2B, and Input were ∼99%. These aligned reads were further processed using SAMtools where unaligned reads were filtered out followed by the sorting of the alignment files, and their conversion to BAM file was performed. Using Input DNA as control, Peak calling was performed with Model-based Analysis of ChIP-Seq (MACS 2.1.3 version) (Zhang et al., 2008). Statistically significant peaks were filtered based on *p*-value cutoff <0.05. The peaks were annotated to the nearest gene or Transcription Start Site (TSS) using HOMER (annotatepeaks) (Heinz et al., 2010). The length distribution of the statistically significant peaks (*p*-value < 0.05) indicates a range of 100–1,000 with majority of them being approximately 100–400. We have used BedSect V3 tool for peaks classification into common and unique peaks across each sample (Mishra et al., 2020).

### Downstream Analysis

Locus overlap analysis (LOLA) was done for the enrichment of annotated peaks over the genomic features and the distance from TSS, and visualized using the GenomicDistribution package in the LOLAweb tool, which is based on LOLA R package (LOLAweb version: 2ddd6d682) (Nagraj et al., 2018). Assessment of TH2B-associated regions with respect to TSS was done by GREAT. MOTIF discovery was performed on fasta sequences of the peak file using MEME SUITE (Bailey et al., 2009) and the significant motifs (E-value ≤ 0.05) were identified. Subsequently, to assign the possible role to these motifs, GOMO was used (Buske et al., 2010). For visualization of coverage depth across the chromosomes, visualization of sorted BAM files was done in integrated genome browser (IGB; https://bioviz.org/) (Nicol et al., 2009). Relative density of coverage across chromosome 1, mm10 was visualized. The *Y*-Axis Scale (0–30) was the same for both TH2A and TH2B graph tracks for comparisons. Heatmaps of different BigWig coverage ChIP signals for sperm PTMs (from GSE79227) on TH2B peaks were generated using Deeptools 3.3.2, computeMatrix (computeMatrix 3.3.2), and plotHeatmap (plotHeatmap 3.3.2) function in Galaxy server (Galaxy Tool Version:3.3.2.0.1) (Blankenberg et al., 2010). Mouse sperm RNA seq data were taken from spermbase (Schuster et al., 2016). All the Venn diagrams have been prepared using BioVenn (Hulsen et al., 2008).

### ChIP-Atlas Public ChIP Datasets Integration and visualization.

Using the peak browser function in ChIP-Atlas (http://chip-atlas.org), peaks associated with primordial germ cells (PGCs), spermatogonial stem cells (SSC), round spermatids, and sperm with a threshold significance of more than 200 were viewed on IGV Ver 2.8.13 (Oki et al., 2018). For prospermatogonia, the maximum available threshold set was 50 and it was thus used for analysis. BigWig coverage tracks for the most significant PTM were retrieved for “mm10” and visualized in IGV. Sets used (as per threshold significance) were GSM1129744 (PGC H3K4me3), GSM1516997 (SSC H3K4me3), GSM1519004 (H3K9ac round spermatid), GSM1046832 (Sperm H3K4me3), and GSM1046834 (Sperm H3K27me3). For comparison, the data range was set the same for all tracks.

### Quantitative Polymerase Chain Reaction (qPCR)

The enrichment of gene regions identified to be associated with TH2A/TH2B by ChIP assays were confirmed by quantitative real-time PCR in triplicate. MACS2 peak specific primers were generated for TH2A-associated genes—*2610005L07Rik, Tcte2, Mt-co1,* and *Mt-tp*—and TH2B-associated genes—*Adam1a, HoxA9, Rec8, Sox30, Nectin2, Dhh,* and *Mxi1* (Supplementary Table S1). Input was prepared from 10% chromatin. Clean-up and concentration of input and ChIP DNA were performed using the ChIP DNA Clean and Concentrator Kit (Zymo Research, Cat #D5205) after overnight proteinase K treatment. PCR reactions contained 1.25 μl of ChIP or input DNA, 5 μmol each of forward and reverse primers, and 12.5 μl of iTaq™ universal SYBR^®^ Green Supermix (Bio-Rad, Cat #1725121) in 25 μl of total volume. Two individual 10-μl reactions were set up in CFX96 Touch Real-Time PCR Detection System (Bio-Rad). The PCR conditions were as follows: 95°C for 3 min; 95°C for 30 s, appropriate annealing/extension and plate read for 30 s for 40 cycles, and melt curve analysis 75–95°C at 0.5°C increment at 5 s/step.

ChIP DNA enrichment was calculated as the percent of input (i.e., the relative amount of IP samples in comparison to 100% input DNA post qPCR) using the formula:

100*2^ (Adjusted Input—Ct (IP))

### Gene Ontology (GO)

Genomic Regions Enrichment of Annotations Tool (GREAT) (http://bejerano.stanford.edu/great/public/html/index.php) version 4.0.4 was used for functional analysis of TH2A/TH2B-associated genomic regions (*p*-value < 0.05) for Mouse: GRCm38 (UCSC mm10, Dec. 2011) (McLean et al., 2010). The default settings were employed. For gene-list-based Gene Ontology, GOliath (GOliath Gene Ontology Search System, Version 0.1.devel) was used. Genes as well as synonyms were identified using Mouse Mine at Mouse Genome Informatics, and this gene list was submitted to GOliath. The chromosome distribution graph was used to calculate the percentage of TH2A/TH2B-associated genes across genome.

### Histone protein sequence retrieval, pairwise alignment, and hydrophobicity map.

Protein FASTA sequences for the following canonical and testis-specific histone variants were retrieved from HistoneDB2.0 (https://www.ncbi.nlm.nih.gov/research/HistoneDB2.0/):

Human TH2A *CURATED SEQUENCE: Homo|25092737|H2A.1 Homo_H2A.1_25092737*, Canonical Human H2A *CURATED SEQUENCE: Homo|10645195|canonical_H2A Homo_canonical_H2A_10645195*.

Canonical H2A mouse: *CURATED SEQUENCE: Mus|30061393|canonical_H2A Mus_canonical_H2A_30061393,* TH2A mouse: *CURATED SEQUENCE: Mus|28316756|H2A.1 Mus_H2A.1_28316756*.

Human Canonical H2B: *CURATED SEQUENCE: Homo|20336754|canonical_H2B Homo_canonical_H2B_20336754,* Human TH2B: *CURATED SEQUENCE: Homo|24586679|H2B.1 Homo_H2B.1_24586679*.

Mouse canonical H2B: *CURATED SEQUENCE: Mus|28316760|canonical_H2B Mus_canonical_H2B_28316760,* Mouse TH2B: *CURATED SEQUENCE: Mus|28316750|H2B.1 Mus_H2B.1_28316750*.

Pairwise global sequence alignment was calculated and visualization was done using Jalview software (Version: 2.11.1.4). Coloring of the residues according to the hydrophobicity was also performed using Jalview (Waterhouse et al., 2009).

## Results

### Pairwise Alignment of TH2B and TH2A in Human and Mouse

Histone variants differ from their replication-dependent canonical counterparts in terms of protein sequences and domains. This difference in sequences may ultimately lead to recruitment of entirely new post-translational modifications (PTMs) and novel interacting chromatin modeling enzymes or modifiers and consequently an altogether new epigenetic profile as compared to that of the somatic histone. Thus, it becomes crucial to closely study the amino acid sequences of these variants. Also, since mice is an experimental model for mammalian studies, to understand the evolutionary conservation of the variants TH2B and TH2A, their homology in protein sequences was assessed by pairwise global alignment of TH2B and TH2A in human and mouse. This revealed ∼95% identity between human and mouse TH2B and ∼89% homology between amino acid sequence of human and mouse TH2A, which differ mostly at the C-terminal (Figures 1B,C). Within the protein sequence, TH2A and H2A have discrepancies at both N- and C-terminal, while TH2B and H2B differ mostly at N-terminal residues in human and mice (Supplementary Figure S1). It is important to note that the N-terminal tails are most crucial for PTMs. UniProtKB analysis for PTMs on the residues different between TH2B and H2B revealed no new PTM (data not shown). However, as the number of hydrophobic residues in the N-terminal region of TH2B is more compared to those in H2B in both humans and mice, this may likely affect the protein–DNA interaction and thermodynamics (Figure 1D).

### Epigenetic Profiling Across the Murine Male Germ Cell Stages for *hist1h2aa* and *hist1h2ba*

**FIGURE 2:**
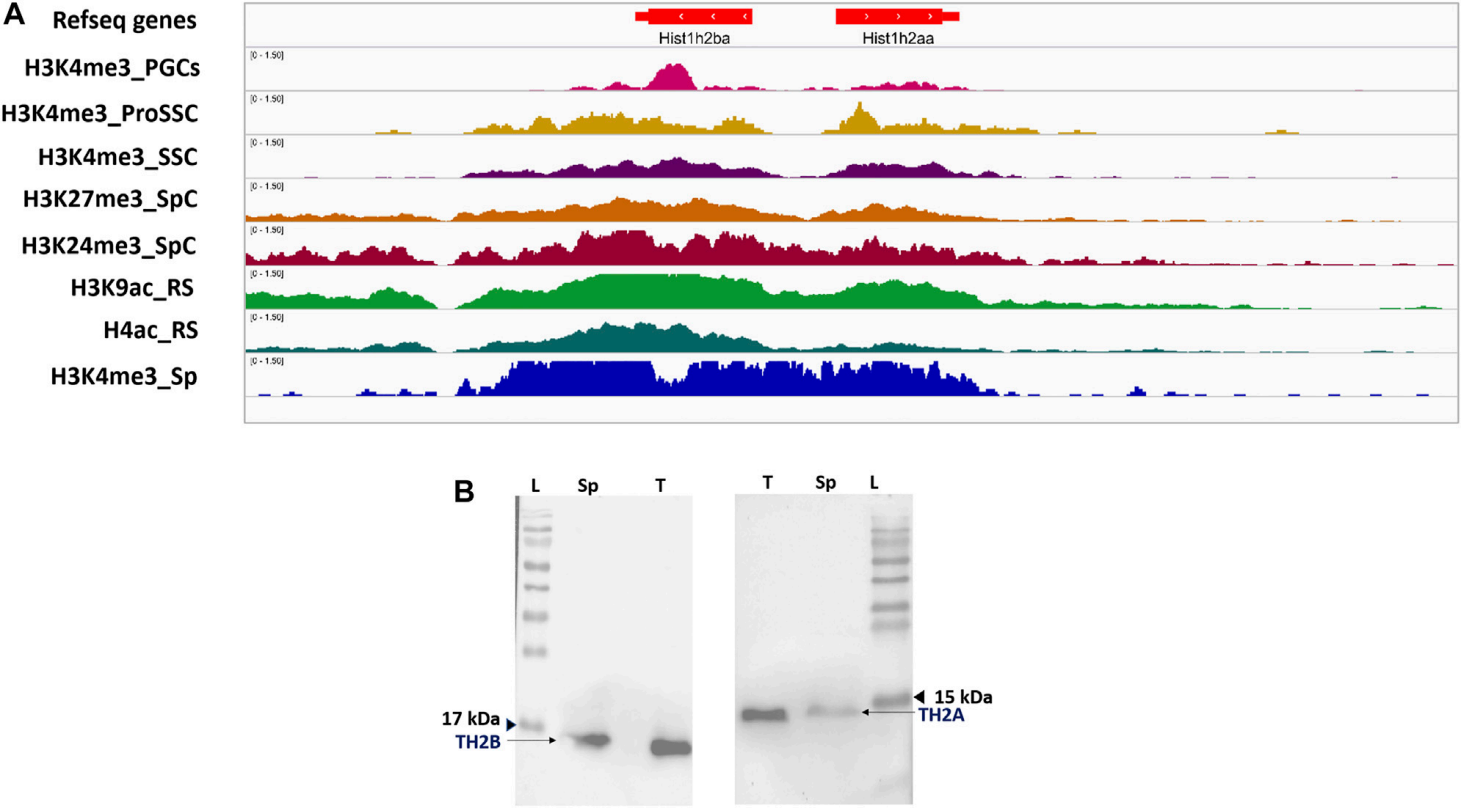
TH2A and TH2B are retained in sperm. **(A)** IGV genome browser view for alignment of ChIP-Atlas bigWig track for post-translationally modified histones at the murine *hist1h2aa* and *hist1h2ba* locus across Primordial germ cells and subsequent male germ cell stages. **(B)** Western blot showing presence of TH2A and TH2B in the mature caudal sperm (Sp) and whole testicular cell lysate (T); protein molecular weight marker (L).

TH2A/TH2B have been reported to be expressed in oocyte, zygote, and testicular germ cells involving spermatocytes and undifferentiated spermatogonia stem cells (SSCs) (Shinagawa et al., 2014; Meistrich et al., 1978; Choi and Chae, 1991; Beedle et al., 2019). We therefore attempted to get an integrative insight into the TH2A/TH2B gene locus to correlate and understand the expression of these histone variants in adult/neonatal germ cells, as well as in prenatal germ cells like PGCs and spermatogonial precursor, prospermatogonia. *Hist1h2aa* and *Hist1h2ba* genes were epigenetically profiled across the murine male germ cell stages, starting from PGCs to sperm using multiple ChIP-seq data from ChIP atlas (Oki et al., 2018). Only highly significant MACS2 peaks were used for the analysis and visualization of bigWig coverage tracks was done using Integrative Genomics Viewer (IGV) under genome assembly mm10 (Robinson et al., 2011). ChIP-seq data analysis shows that murine TH2A and TH2B genes are associated with activating mark H3K4me3 in PGCs, prospermatogonia (PRO_SSC), spermatocytes (SpC), and sperm (Sp) (Figure 2A). In the round spermatids (RS), TH2A/TH2B are marked by H4ac and H3K9ac, the latter being a mark of active promoters. Enrichment is mostly restricted to the *hist1h2aa* and *hist1h2ba* suggestive of active expression of their cognate proteins TH2A and TH2B, respectively, across the germ cell stages. This epigenetic PTMs map also supports the already published data for SSCs, spermatocytes, and spermatid expression of TH2A/TH2B (Meistrich et al., 1978; Choi and Chae, 1991; Shinagawa et al., 2014; Beedle et al., 2019).

Western blotting also depicts the presence of these histones in the caudal sperm as well as testis (Figure 2B). Furthermore, in order to cross-validate our finding, a similar analysis was done for other testis-specific histone variants *H3f3b* and *H1fnt*. A profile similar to that of TH2A/TH2B was found for H3f3b (H3.3) whereas H1fnt, which is involved in spermiogenesis, predominantly showed H4ac and H3K9ac marks in round spermatids and H3K4me3 in sperm (Supplementary Figures S2A,B). This correlates with the expression dynamics of H1fnt (Shalini et al., 2021).

It is well known that up to spermatids, the nucleohistone organization in the male germ cell is similar to that of any somatic cell in the body. It has also been established that the presence of TH2B in spermatids is important for nucleohistone-to-protamine transition (Montellier et al., 2013). That TH2A/TH2B destabilizes the nucleosome has also been documented (Padavattan et al., 2015). However, even after histone replacement, the mature sperm still retain these variants. Thus, to demystify the importance of their retention, we did genome-wide mapping of these two histone variants in the caudal sperm.

### Genome-wide Distribution of TH2A and TH2B in Sperm

**FIGURE 3:**
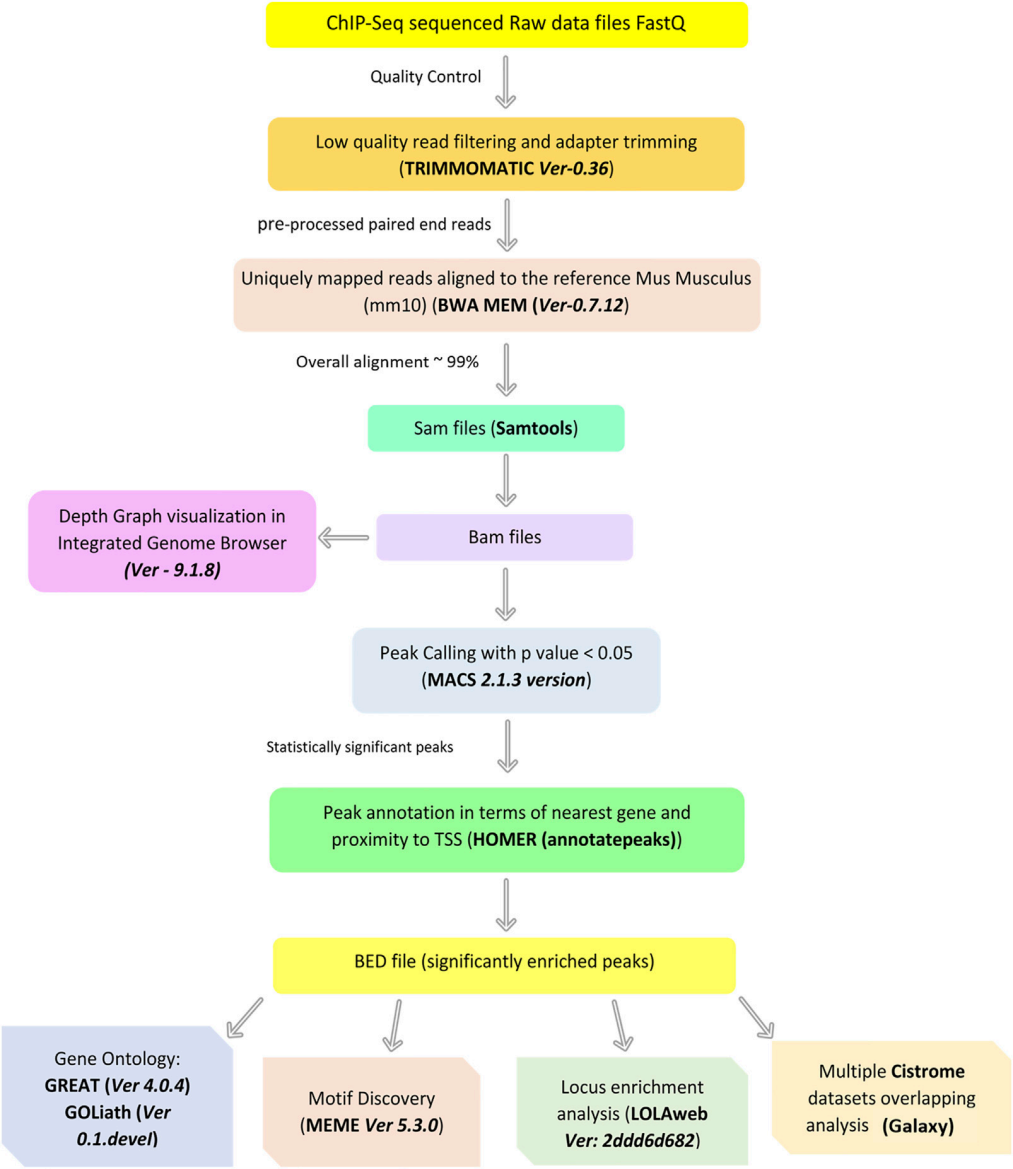
Brief summary of the ChIP-Seq data processing and downstream analysis undertaken in this study.

The genomic distribution of TH2A/TH2B in mature sperm was explored using chromatin immunoprecipitation sequencing (ChIP-Seq). A flow chart depicting a brief summary of the bioinformatics analysis is shown in Figure 3.

**FIGURE 4:**
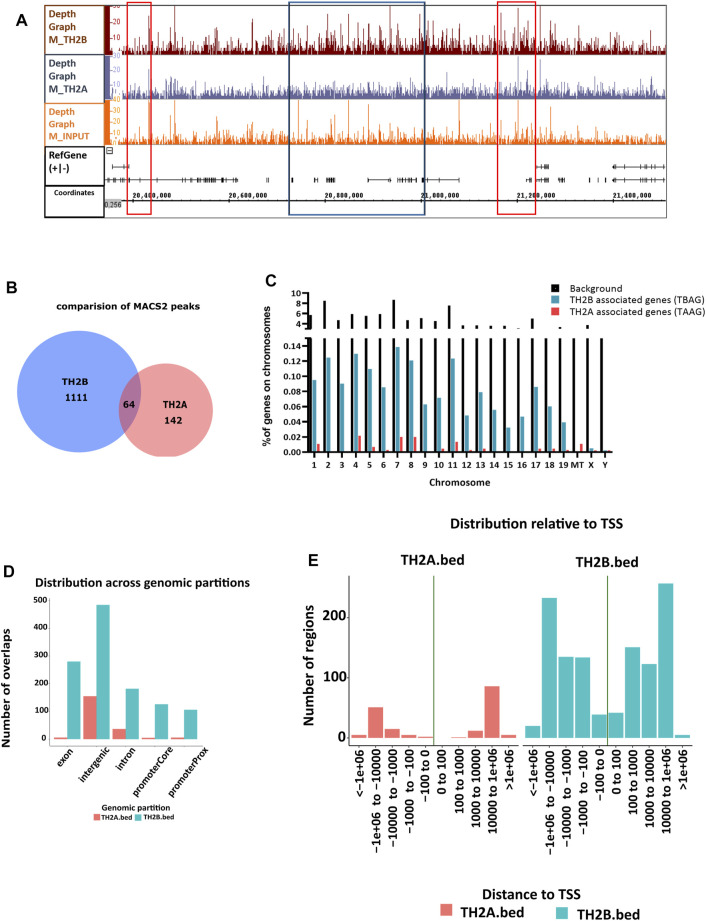
Genome-wide distribution of TH2A and TH2B in sperm. **(A)** Integrated Genome Browser view of relative coverage for chromosome 1 (mm10) as shown by depth graph using sorted BAM files for TH2A, TH2B, and Input. The red box depicts regions of similarity between TH2A and TH2B profiles while the blue box denotes the differences. **(B)** Venn diagram showing the common and unique MACS peaks between TH2A and TH2B. **(C)** Bar chart describing the percentage of gene occupancy per chromosome for TH2A (red bars)- and TH2B (blue bars)-associated genes with respect to total genes on that chromosome, background (black bars). **(D)** TH2A and TH2B distribution across genomic partitions. **(E)** Relative distance of TH2B and TH2A MACS2 peaks from the transcription start site (TSS).

In all, 1,180 peaks were identified to be associated with TH2B and 211 peaks or genomic regions were identified to be associated with TH2A. Depth graph coverage visualization (representative image for chromosome 1) of binary alignment files in Integrated Genome Browser (IGB) (Nicol et al., 2009) revealed regions of similarity and differences between TH2A and TH2B coverage (Figure 4A). Comparison of MACS2 peak sets for the two histone variants revealed 64 peaks common between the two (Figure 4B). This is equivalent to ∼30% peaks of TH2A but roughly 5% of TH2B peaks. As TH2A and TH2B in a nucleosome poise for an open chromatin which could likely be indicative of active transcription of associated genes, we looked for any peaks shared by these histone variants in the transcription start site (TSS). Overall, 16 common TSS peaks were found (Supplementary Figure S3A). Next, we calculated the genome-wide coverage of TH2B-associated genes (TBAG) and TH2A-associated genes (TAAG), using GOliath (www.bioinformatics.babraham.ac.uk/projects/goliath/). A chromosome wise coverage map was generated (Figure 4C). Considering the mouse genome to be composed of 50,083 genes on the basis of genes with nucleotide sequence data in Mouse Genome Informatics (Goldsmith et al., 2005), an occupancy of ∼1.6% TH2B-associated genes and 0.14% TH2A-associated genes was seen on the chromosomes. This percentage is within the reported range of histone retention in murine sperm, i.e., 1%–10%.

TH2B association was noted on all autosomes and sex chromosomes except the mitochondrial (MT) chromosome. However, unlike TH2B, a staggered association of TH2A binding loci with genic regions was seen, with binding sites only on chromosomes 1, 4–8, 10–13, 17–19, MT, X, and Y. A fair enrichment was seen on MT chromosome. Locus enrichment overlap analysis was done using LOLAweb (Nagraj et al., 2018). TH2A- and TH2B-associated genomic intervals showed significant overlaps with intergenic and intronic regions (Figure 4D). Approximately 35% of the TH2B-associated peaks were detected within ±5 kb of TSS (Supplementary Figure S3B). Importantly, majority of the TH2B-associated regions covered the exon as well as the core and proximal promoter. As also evident from the genomic partition (Figure 4D), a significant number of TH2B peaks were detected within ±1 kb of TSS, whereas TH2A peaks distribution at TSS was sparse (Figure 4E). TH2A and TH2B peaks were subjected to MEME ChIP software to identify the significant motifs recurring in the peak sequences. The three most significant sequences are tabulated (Table 1, Table 2). Additionally, we associated Gene Ontology to the discovered motifs using GOMo (version:5.3.0) (Bailey et al., 2009). A motif CTGTGGTGTCACAGT common to TH2A and TH2B was discovered. It is similar to Cyclic AMP-dependent transcription factor (AtF1). This gene regulates a number of physiological processes and is thus quite universal in binding. Commonality of the motif might be due to the overlapping peaks between the two histone variants. Thus, TH2B encompasses a wider genomic localization across all autosomes including promoter and exonic regions on genes as compared to the relatively sparse genomic association of TH2A, which is predominantly intronic or intergenic.

**TABLE 1:** Significant motifs in TH2A sequences.

Logo	E-value	Sites	Known or similar motifs	GOMo
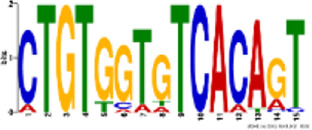	2.5e-046	20	Atf1_primary (UP00020_1)	BP sensory perception of smell; G-protein-coupled receptor protein signaling pathway; signal transduction; cell communication MF olfactory receptor activity
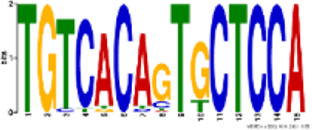	8.5e-068	22	None	BP immune response; G-protein-coupled receptor protein signaling pathway; response to external stimulus; sensory perception CC plasma membrane
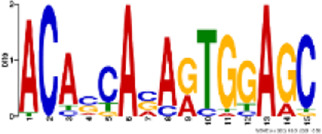	5.9e-023	22	None	BP sensory perception of smell; G-protein-coupled receptor protein signaling pathway; filopodium assembly CC integral to membrane MF olfactory receptor activity

**TABLE 2:** Significant motifs in TH2B sequences.

Logo	E-value	Sites	Known or similar motifs	GOMo
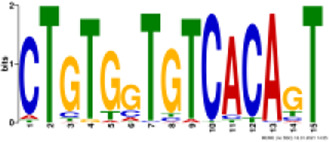	1.2e-100	56	Atf1_primary (UP00020_1)	BP sensory perception of smell; G-protein-coupled receptor protein signaling pathway; signal transduction; cell communication MF olfactory receptor activity
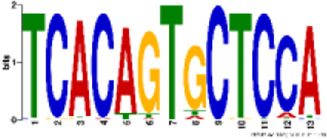	2.2e-077	53	Zfp691_primary (UP00095_1)	BP sensory perception of smell; G-protein-coupled receptor protein signaling pathway; signal transduction CC integral to membrane MF olfactory receptor activity
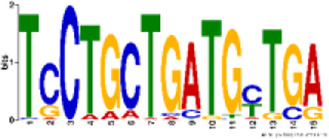	2.9e-081	46	None	CC integral to membrane CC extracellular space MF calcium ion binding; transmembrane receptor activity; serine-type endopeptidase activity

BP—biological process; CC—cellular component; MF—molecular function.

### Gene Ontology Analysis for TAAGs and TBAGs

**FIGURE 5:**
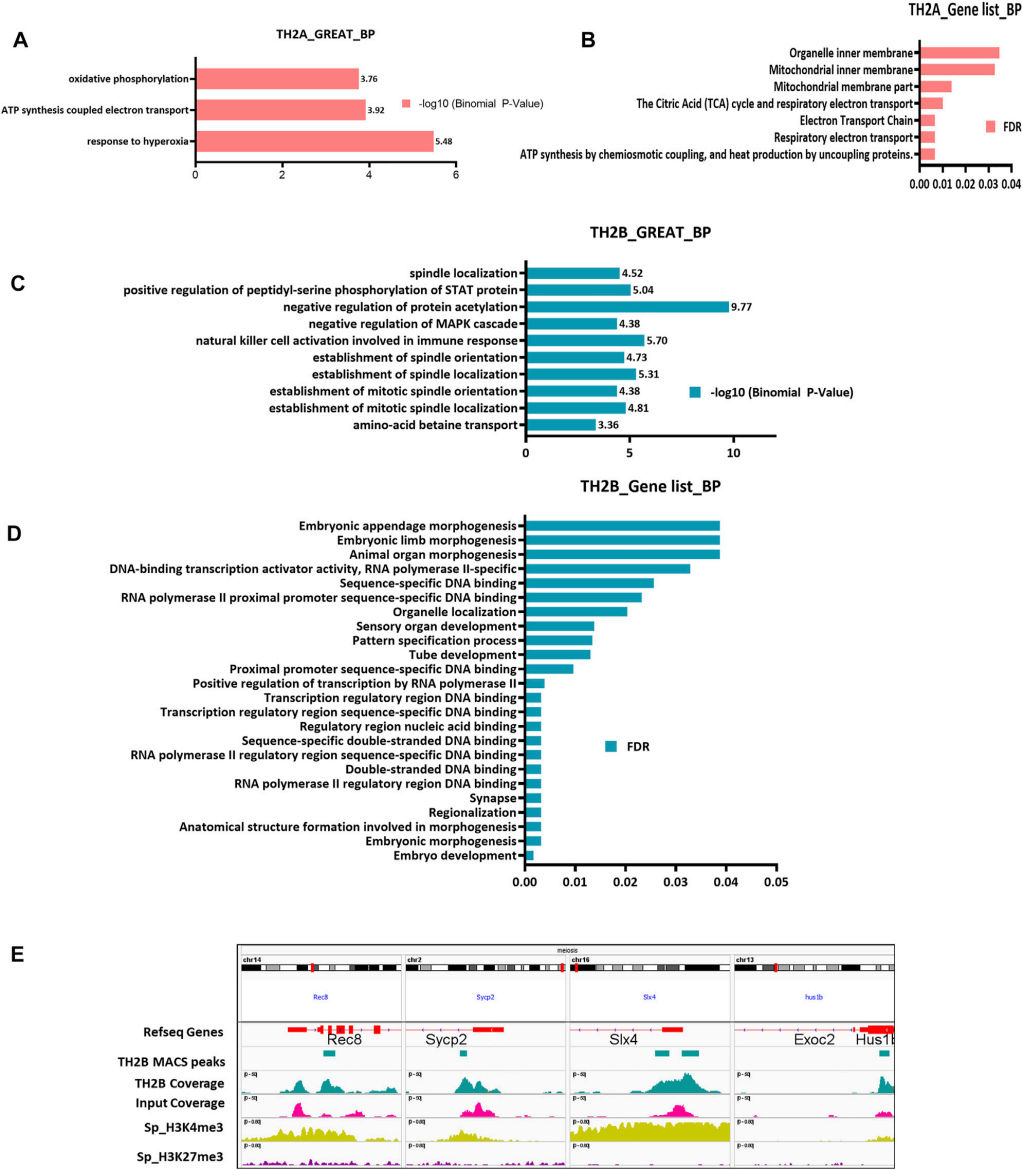
Genomic intervals as well as gene list-based Gene Ontology (GO) enrichment analysis. **(A)** Genomic region-based Gene Ontology (Biological processes) for TH2A bound chromatin using GREAT tool calculated according to–log10 (Binomial *p*-value). **(B)** Gene Ontology-Biological Processes for TH2A-associated genes using GOliath ranked as per the false discovery rate (FDR). **(C)** Genomic region-based Gene Ontology (Biological processes) for TH2B-bound chromatin using GREAT tool calculated according to–log10 (Binomial *p*-value). **(D)** Gene Ontology-Biological Processes for TH2B-associated genes using GOliath ranked as per the false discovery rate (FDR). **(E)** IGV view showing TH2B MACS peak at genes important for meiosis—Rec8, Sycp2, Slx4, and Hus1b along with ChIP Atlas bigWig coverage alignment tracks for sperm PTMs H3K4me3 (activating mark) and H3K27me3 (repressive mark).

Having mapped the histone variants retained in the mature sperm, characterization of their associated loci/genes was performed to understand the significance of their retention. Genomic regions enrichment of annotations tool (GREAT; to understand the *cis*-regulatory interactions of TH2A/TH2B with their associated loci) and GOliath (gene list-based Gene Ontology) were used for Gene Ontology analyses of the genomic regions for TBAG and TAAG lists (Figure 5). For TH2A, “ATP synthesis”, “Electron Transport Chain”, and “oxidative phosphorylation” emerged as the most significant terms (Figures 5A,B). This observation is in sync with the detection of TH2A association with mitochondrial genes.

For TH2B, GREAT tool identified terms like “negative regulation of protein acetylation and MAPK cascade” apart from “positive regulation of peptidyl serine phosphorylation of STAT protein” (Figure 5C). These terms indicate gene inactivation by negatively regulating protein acetylation and MAPK, as well as transcription activation by serine phosphorylation of Signal Transducer and Activator of Transcription (STAT) protein. Thus, it may represent a mechanism of transcription regulation during spermiogenesis wherein, at the round spermatid stage, there is a burst of transcription followed by systematic shutdown of transcription machinery as the spermatid matures into sperm. Also featuring in the list was “amino acid betaine transport”, which is known to improve spermatogenesis (Shadmehr et al., 2018).

Importantly. “spindle assembly” associated terms featured in 5 of the top 10 significant ontology searches (Figure 5C). Proper spindle assembly is crucial for segregation of chromosomes during meiosis and mitosis. Spermatids of TH2A/TH2B double knock out (KO) mice show incomplete cohesin release and thus disruption of chromosome segregation, along with retention of meiosis-specific proteins like rec8 (REC8 meiotic recombination protein) and scp3 as a result of which the male knockout mice are infertile. In light of these observations, our finding of TH2B association with spindle assembly assumes importance. With GOliath, for TBAG, the term “embryo development” showed the lowest False Discovery Rate (FDR) of 0.001684 (Figure 5D). Apart from embryo development, a number of hits were noted with respect to RNA polymerase II proximal promoter and regulatory region DNA-binding (Figure 5D). These may be attributed to TH2B being a histone protein that promotes open chromatin architecture and hence assisting RNA pol in transcription. Also, as previously stated, ∼35% of TH2B peaks were within 5 kb of TSS.

As spindle organization featured prominently, TH2B association with meiosis-specific genes was investigated. TH2B was found to be associated with meiosis genes *Lemd2, Mov10l1, Ndc1, Psmd13, Rec8, Slx4, Syce1*, and *Sycp2* (Figure 5E). Ndc1 is required for association of chromosomes to spindles for proper segregation to daughter cells (Thomas and Botstein, 1986). Lemd2 is a nuclear lamina protein critical for nuclear envelope integrity and reported to regulate MAP and AKT kinase (Ulbert et al., 2006; Tapia et al., 2015). It is also reported in the spermatid and might be involved in nuclear remodeling (Elkhatib et al., 2017). MOV10L1 is a protein crucial for male fertility, which protects spermatocytes from retrotransposons activation. MOV10L1 knock outs show complete abrogation of spermatogenesis at meiosis 1 prophase (Frost et al., 2010). *Slx4* (SLX4 structure-specific endonuclease subunit) is an endodeoxyribonuclease, important for the resolution of Holliday Junctions (HJs), which is essential for segregation of chromosomes. Yeast *Slx4*’s mammalian ortholog, BTBD12 mutants are sub-fertile with almost under 15% normal spermatozoa (Holloway et al., 2011). It is also involved in double-stranded break repair during recombination events. *Sycp2* (Synaptonemal Complex Protein 2) is an important meiotic synaptonemal complex protein part of axial elements/lateral elements. Male mutants of *Sycp2* have defects in meiosis with failure in axial element formation and are sterile (Yang et al., 2006). ChIP-Seq also revealed TH2B bearing nucleosomes to lie in the exonic region of HUS1 checkpoint clamp component B (*Hus1b*) cell cycle checkpoint gene required for genomic integrity (Hang et al., 2002). Also, after profiling the epigenetic association for four of these genes, we found H3K4me3 but not the repressive H3K27me3 mark association with TH2B (Figure 5E). Exclusive of these genes, TBAGs also include other genes important for chromosome segregation like *Cep57l1, Esco2, Hjurp, Ncapg, Rrs1, Top3b, Trappc12,* and *Ube2i* (Milev et al., 2015; Ivanov et al., 2018; Zhang et al., 2019; Ito et al., 2021)*.* This evidence provides an insight into the putative role of TH2B in meiosis and also understands the physiology of TH2B/TH2A KO mice.

**FIGURE 6:**
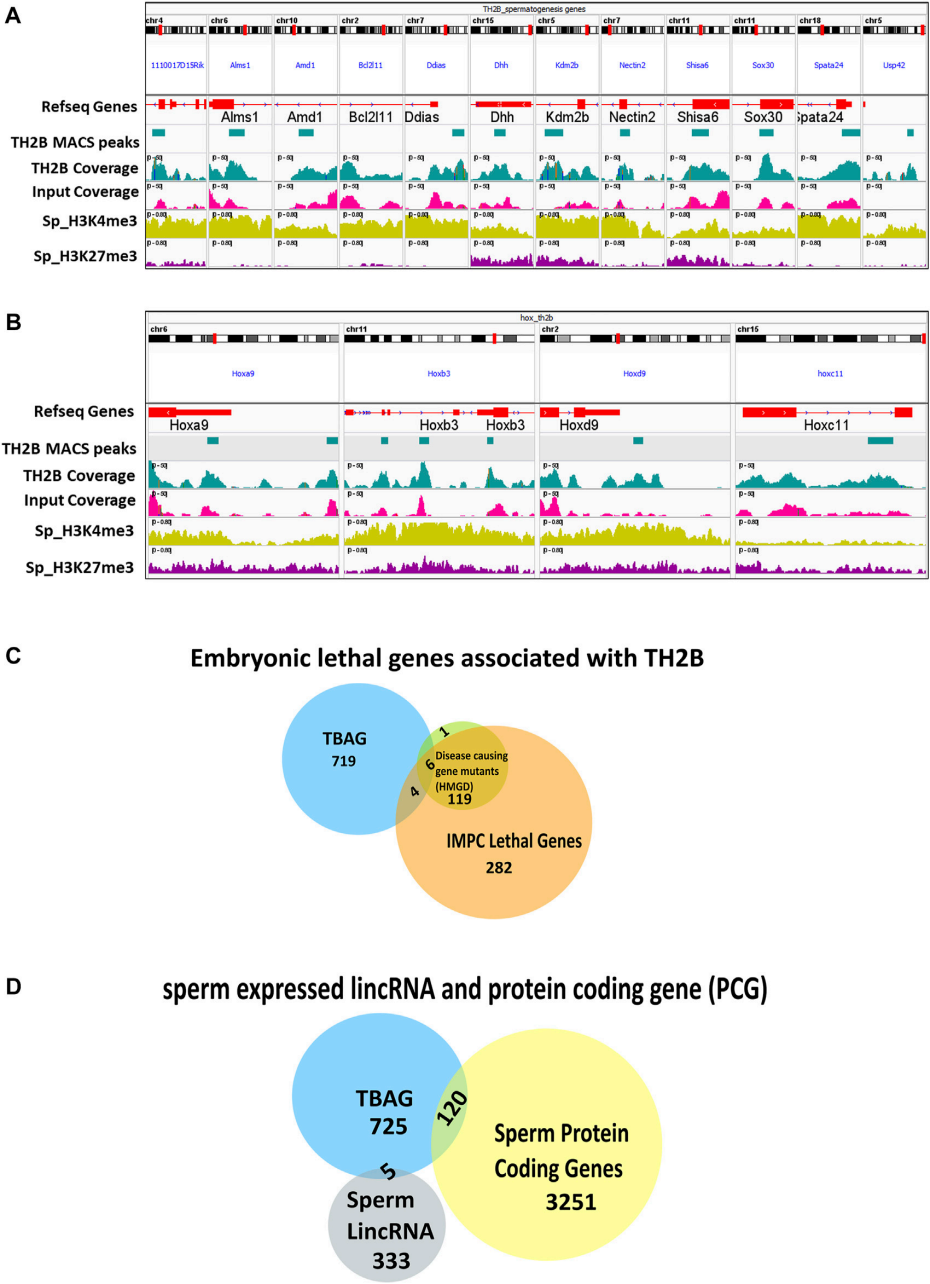
**(A, B)** IGV view showing TH2B MACS2 peak at genes important for spermatogenesis and at HOX cluster genes, respectively, along with ChIP Atlas bigWig coverage alignment tracks for sperm PTMs H3K4me3 (activating mark) and H3K27me3 (repressive mark). **(C)** Venn diagram showing the common embryonically lethal genes between TH2B-associated gene (TBAG) list and gene lists extracted from the International Mouse Phenotyping Consortium (IMPC) and Human Genome Mutation Database (HGMD). **(D)** Venn diagram showcasing the TBAGs common to sperm-derived protein coding gene (PCG) list and lincRNA list.

Apart from the meiosis-specific genes, a number of spermatogenesis specific genes were also identified to be associated with sperm-retained TH2B. These include *1110017D15Rik, Alms, Amd1, Bcl2l11, Ccin, Ddias, Dhh, Gli1, Gopc, Gopc, H1f7, Hps1, Kdm2b, Larp7, Meig1, Mov10l1, Ndc1, Nectin2, Neurl1a, Poc1a, Shisa6, Sox30, Spata24*, and *Usp42* (Details in Supplementary Table S2). Most of these genes are important for spermatid development, spermatid nuclear condensation, and axoneme assembly. In light of the knowledge that during spermiogenesis there is a surge in transcription of spermiogenesis-specific genes, TH2B association with these loci justifies that this histone variant escapes protamination in order to be accessible for the final spermatogenic events. Integrated Genomics Viewer visualization for 11 of these genes with TH2B peaks is shown in Figure 6A. Most of the genes showed the absence of H327me3 mark. Previous reports indicate that H3K4me3/H3K4me2 are abundantly present in mouse sperm and both the marks have overlapping regions in the genome (Brykczynska et al., 2010; Erkek et al., 2013; Siklenka et al., 2015). Spermatogenesis-related genes are marked by tri- or di-methylated H3K4. Our observation of depletion of the repressive mark on most of the TH2B-associated genes is in concordance with these reports.

The coverage of TH2A/TH2B at spermatogenically important protamine locus and *tnp2* gene was also examined. In comparison to TH2A, TH2B had significant coverage at the *Prm1, Prm2, Prm3*, and *Tnp2* genes with respect to Input (Supplementary Figure S4A). Recently, a group identified 54 evolutionary conserved testis enriched genes that are not essential for fertility in males (Miyata et al., 2016). Of the 54 genes, we found only three genes: *RIKEN cDNA 1700011E24* gene (*1700011E24Rik*)*, RIKEN cDNA 1110017D15* gene (*1110017D15Rik*), and testis, prostate, and placenta expressed (*Tepp*) (Supplementary Figure S4B). This suggests that TH2B was associated with majority of the genes important/essential for male fertility.

Evident from the gene list-based Gene Ontology enrichment for TBAG, least FDR for the term “embryo development” indicated the importance of sperm-retained TH2B in embryo development. Also, as already mentioned for their contribution in spermatogenesis, TBAGs like *Tgif1, Mxi1, Zfp830, Thbd, Ppard, Dlk1*, and *Asf1b* are also involved in various aspects of embryogenesis like blastocyst growth, development and hatching, embryo implantation, and gastrulation. From their pioneer study using sperm from fertile human donors, Hammoud et al. have shown that the retention of histones is not random but on loci of developmental importance, imprinted genes, and regulatory RNAs (Hammoud et al., 2009). Consequently, we investigated whether TH2B was associated with developmentally important loci like HOX cluster. Contrary to the findings of Hammoud et al. (2009) in human sperm where they did not find TH2B enrichment on developmentally important genes, our data indicated the presence of TH2B on all four HOX clusters—*Hoxa, Hoxb, Hoxc*, and *Hoxd* (Figure 6B). Similar to the observations of Brykczynska and coworkers (Brykczynska et al., 2010), these TH2B-associated Hox loci show bivalency in terms of H3K4me3 and H3K27me3. This is important for “poising” these genes for active transcription post fertilization (Hammoud et al., 2009, 2014).

As TH2B also marks a developmentally important Hox gene cluster that harbors bivalent marks, we extended our search to embryonic lethal gene. Towards this, the data for 410 murine lethal genes from the International Mouse Phenotyping Consortium (IMPC) were referred (Dickinson et al., 2016). Four embryonic lethal genes—*4933427d14rik, Aim1, Asf1a*, and *Slc39a8*—were identified to be associated with TH2B. As mouse embryonic lethal genes may likely have a human disease phenotype, TBAGs were also inspected against the Human Genome Mutation Database (HGMD) and IMPC data. This search yielded six TBAGs—*Epas1, Epc2, Lmbrd1, Msx1, Pitx2*, and *Slc40a1* (Figure 6C). Moreover, to identify any sperm-specific lincRNAs and/or protein coding gene (PCG)-related sperm chromatin signatures (here TH2B associated), we took the dataset filtered by Subhash et al. who have reported that bivalency at sperm-specific PCGs are maintained throughout development and also sperm-specific lincRNAs reach their peak expression at the time of zygotic genome activation (Subhash et al., 2020). One hundred twenty sperm protein coding genes and five lincRNAs were identified to be associated with TH2B (Figure 6D). Four TH2B-linked regulatory RNAs, namely, *Mir219a-2, Mir196b, Kcnq1ot1*, and *1010001N08Rik*, were also present. Of these, miRNA *miR196b* has been reported to be enriched in the mouse testis while *miR219a-2* was barely detected in the testis (Isakova et al., 2020). These miRNAs were associated with activating modification H3K4me3 in sperm (Supplementary Figure S5).

**FIGURE 7:**
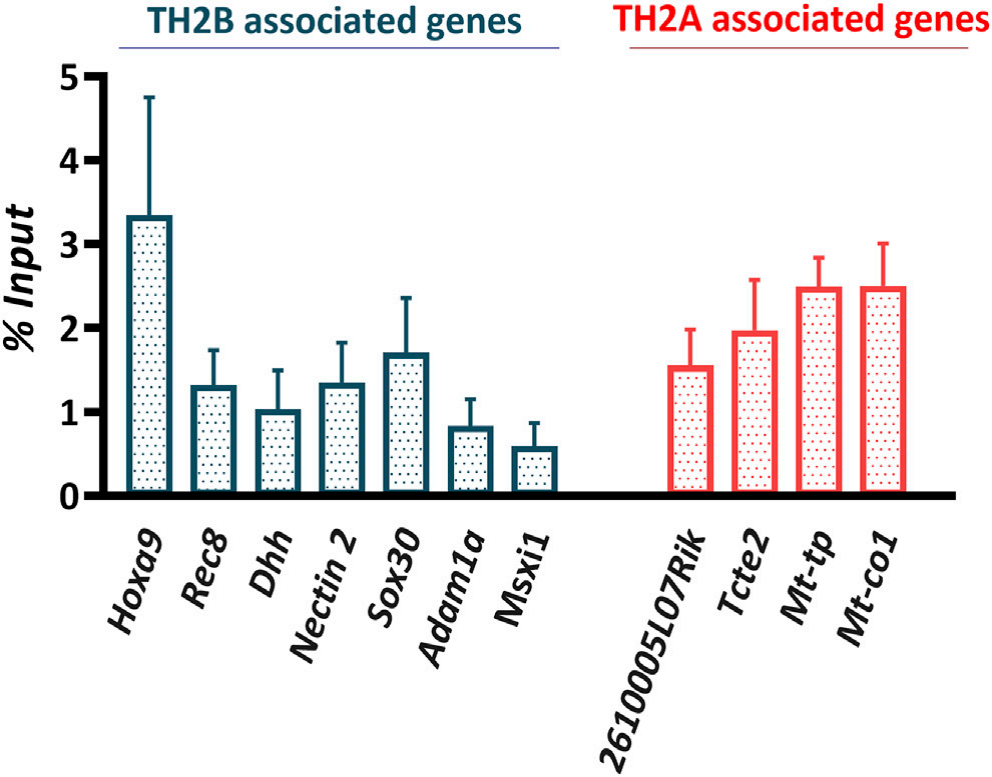
ChIP assay validation by ChIP qPCR represented as percent input. Bars represent standard error of mean (SEM).

A set of TBAGs—*Hoxa9, Rec8, Dhh, Nectin2, Sox30, Adam1a,* and *Msxi1—*and TAAGs—*2610005L07Rik, Tcte2, Mt-tp,* and *Mt-co1*—were cross-validated by ChIP-qPCR (Figure 7). These findings impart significance to the ∼2% TH2B retained in mature sperm, with respect to spermatogenesis and post-fertilization embryonic events.

### PTMs Associated With TH2B Occupied Loci

**FIGURE 8:**
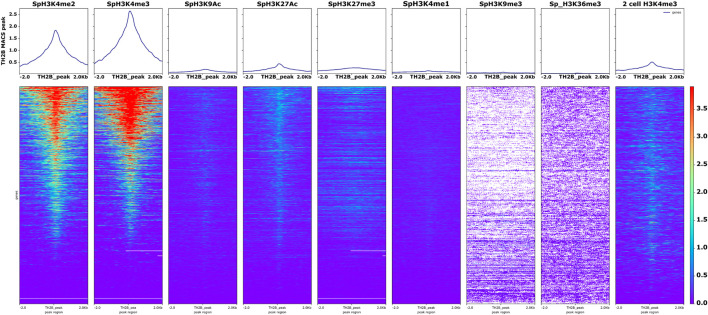
Mature sperm-retained TH2B-associated loci overlap with activating modifications H3K4me3/H3K4me2 in sperm and two-cell embryos but not with the repressive marks in sperm. Heatmap and profile plot for various sperm-associated modified histones and their overlap with reference to TH2B MACS2 peak. Scale on the right-hand side reveals the intensity of overlap scores between the query set (TH2B BED) and sperm PTMs.

The nucleosome consists of two copies each of H2A, H2B, H3, and H4 along with 147 bp DNA and H1 linker histone. TH2B being the major histone variant of H2B in mature sperm, it replaces H2B in ∼85% nucleosomes. The “histone code” is established by a consortium of PTMs that ultimately regulate the expression of genes associated with them. Thus, although a number of loci may be associated with a particular histone, their expression is only guaranteed if they have an activating modification at the histone associated with them. A repressive mark in association may lead to repression or silencing of the said loci. Thus, in order to understand the PTMs like acetylation and methylation on TH2B-associated loci, the murine sperm histone modification datasets of GSE79227 (https://www.ncbi.nlm.nih.gov/geo/query/acc.cgi?acc=GSE79227) were used for identifying activating histone marks (H3K4me3/me2 and H3K9Ac), super enhancer modifications (H3K27Ac and H3K4me1), and repressive marks (H3K27me3, H3K9me3, and H3K36me3). The genome-wide BigWig coverage tracks for mm10 were retrieved using ChIP atlas and these bigWig scores were submitted to Galaxy along with the TH2B genomic interval file. Using the computeMatrix function, a matrix file was generated for signals ±2 kb around the TH2B MACS2 peak. The computed matrix was then visualized using plotHeatmap. While repressive marks H3K27me3, H3K9me3, and H3K36me3 showed no overlap with TH2B chromatin, a positive enrichment of H3K4me2 and H3K4me3 overlapping the peak sequences was observed (Figure 8).

A mild correlation was observed between active enhancer mark H3K27Ac and TH2B marked loci. Recent chromatin analysis studies on sperm have shown that H3K4me2 and H3K4me3 marks are both enriched in sperm, with many overlapping regions marked by these PTMs (Siklenka et al., 2015; Lambrot et al., 2019; Lismer et al., 2020). Thus, our data were subjected to Enhancer Atlas 2.0 (Gao and Qian, 2020). Several testis/spermatid-based enhancers were identified (Supplementary Table S3). Fifteen TH2B-associated enhancers that regulated 112 genes were observed. Transcript level evaluation of these 112 genes was done using mouse sperm RNA-seq data from spermbase (Schuster et al., 2016). Transcripts of 76 of these 112 genes were seen to be present in sperm. We studied the expression profile of the 112 genes across tissues using NCBI gene expression data. Analysis revealed at least five genes (*4921511H03Rik, Speer4e, Iqcf3, Iqcf4*, and *Iqcf6*) with expression restricted to testis, nine genes (*Gm9758, Gm17019, Speer4d, Gm10354, Tmed6, Tmem194, Ggnbp1, Ska1,* and *Ccdc11*) with biased testicular tissue expression, and eight ubiquitously expressing genes (*Zfp956, Vps4a, Cog8, Poc1a, Baz2a, Rnf41, Dynlt1a,* and *2900010M23Rik*) with highest expression in testis. Only three genes (*Sntb2, Dynlt1b,* and *Dynlt1c*) were identified with broad expression across all the tissues and highest expression in testis. Intriguingly, two enhancers (chr5:7179305–7179,545 and chr5:14974466–14974712) controlled the expression of six genes: *4921511H03Rik, Gm9758, Gm10354, Speer4d, Gm17019*, and *Speer4e*, which have either biased or restricted testicular expression.

The TH2B peaks were also scored against the two-cell H3K4me3 dataset. This was based on the evidence of inheritance of paternal epigenome to the zygote and across developmental stages *via* sperm H3K4me3 (Lismer et al., 2020). We found a mild degree of overlap between the sets (Figure 8). More mechanistic studies are needed to understand the paternal transmission of TH2B post fertilization.

### TBAGs in Human and Murine Sperm

**FIGURE 9:**
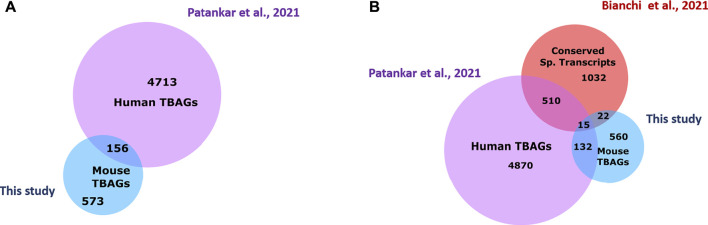
Comparison of human- and murine sperm-retained TH2B. **(A)** Venn diagram focusing on the common and unique genes between the human and murine TH2B orthologs. **(B)** Conserved sperm transcripts common to human and mouse TH2B-associated genes (TBAGs).

*Mus musculus* being an experimental model, comparison of our murine TH2B data was done with our group’s recently published ChIP-Seq TH2B data derived using human sperm (Patankar et al., 2021). Because of the difference in the genomic coordinates between the two assemblies, the annotated MACS peaks were compared based on gene names. Using MGI, the mouse orthologs for the human sperm TBAG were identified and intersected with the murine TBAG list. A total of 156 hits were found common between the two sets (Figure 9A). This is equal to 26% of murine TH2B occupancy as against 3.5% of human TH2B association. The highly significant and the only GO term in MGI for the common genes was “anatomical structure morphogenesis”. Analysis of the common genes revealed 21 genes involved in embryo developmental processes: *Abl1, Col12a1, Epas1, Grem2, Insig2, Invs, Lmo4, Msx1, Myo6, N4bp2l2, Npat, Nr4a3, Osr1, Pax1, Pcsk6, Pitx2, Spry2, Tbx20, Tbx5, Tcf7l2*, and *Zpr1* (Supplementary Table S4). The conserved association of these embryogenesis-related genes with TH2B across two mammalian species highlights the importance of TH2B retention across species. It also highlights that these TH2B loci selectively escape protamination in both the species. These genes will have a relatively more open chromatin and may be transcribed immediately post fertilization or in the early preimplantation stages.

The TBAGs common in human and mouse sperm were also investigated for their functional relevance by analyzing them against sperm function-related transcripts that have been reported to be conserved across human, rat, and mice (Bianchi et al., 2021). Fifteen sperm function oriented conserved transcripts were found common to both human and mouse TBAGs (Figure 9B; Supplementary Table S5). The H3K4me3 status was also checked at these loci using mouse TH2B data. Most of the genes were marked by H3K4me3 modification (Supplementary Figure S6). Thus, TH2B is conserved at many common loci important for spermatogenesis as well as embryogenesis in both human and murine sperm. Also, the systematic association of activating modification with these conserved loci will “poise” them for early transcription during embryogenesis as opposed to random sperm histone retention (Carrell and Hammoud, 2009).

## Discussion

Sperm chromatin is uniquely packaged into protamines with only a few important loci escaping the histone to protamine reprogramming during spermiogenesis. The retained histones include several histone variants that aid in the histone-to-protamine transition by inducing an open chromatin thus promoting histone eviction. TH2A/TH2B are two such histones as revealed by Nucleosomal Core Particle (NCP) studies (Padavattan et al., 2015, 2017). Pairwise alignment of TH2B and TH2A in human and mouse reveals that human and mouse TH2B are more homologous to each other with ∼95% identity as compared to ∼89% homology shared by human and mouse TH2A (Figures 1B,C).

While the importance of TH2A/TH2B in histone eviction is well acknowledged, the significance of their retention in mature sperm is still unclear. The present study attempted to demystify the relevance of sperm-retained TH2A/TH2B.

### Murine *Hist1h2aa* and *Hist1h2ba* Are Associated With Activating Modification H3K4me3 From PGCs to Sperm

TH2A/TH2B are expressed in oocyte, testis, and zygote (Shinagawa et al., 2014). In the testis, incorporation of TH2A/TH2B in the male germ cell chromatin has been shown from spermatocytes onward with a recent report showing the expression in undifferentiated spermatogonia using immunofluorescence (Meistrich et al., 1978; Choi and Chae, 1991; Beedle et al., 2019). Undifferentiated spermatogonia stem cells (SSCs) are successors to prospermatogonia (Niedenberger et al., 2015). In order to understand this expression profile of TH2A/TH2B in terms of the epigenetic landscape, the TH2A/TH2B locus was mapped *in silico via* ChIP-seq data across germ cell stages (Figure 2). This profiling analysis revealed association with activating mark H3K4me3 in PGCs, prospermatogonia (PRO_SSC), spermatocytes (SpC), and sperm (Sp). As open chromatin is also a feature of active gene expression, the presence of activating modification on *Hist1h2aa/Hist1h2ba* in PGCs and pro-spermatogonia might lead to their active gene expression. Since these histones are associated with open chromatin, many pluripotency and multipotency genes in PGCs and pro_SSCs might be marked by them to promote active transcription, an observation similar to that of Beedle and coworkers (Beedle et al., 2019). This observation needs to be further explored with TH2A/TH2B studies in PGCs and prospermatogonia. TH2A/TH2B association with acetylation marks, H4ac and H3K9ac, in the round spermatid (RS) is justified as RS is the germ cell stage where H4 histone hyperacetylation to evict the histones and transcription burst for spermiogenesis-specific genes occurs.

### TH2A and TH2B Exhibit Distinct Epigenetic Profiles

A previous report by Montellier et al. point at the importance of TH2B in the transition of epigenetic state from histones to protamine during spermatid cell stage (Montellier et al., 2013). Padavattan and co-workers have also pointed that TH2A/TH2B induce nucleosomal instability, which helps in histone destabilization (Padavattan et al., 2015). Also, there is a pre-existing belief that TH2A and TH2B work in concert. The presence of activating mark with TH2A/TH2B (Figure 2A) and retention of these histone variants in the mature sperm as revealed by Western blotting analysis (Figure 2B) made it crucial to explore their function in the sperm. To understand this retention, chromatin immunoprecipitation sequencing (ChIP-Seq) was deployed to determine the genome-wide distribution of TH2A/TH2B in sperm. We found significant TH2A chromatin enriched with MtDNA peaks. It was intriguing, as the immunoprecipitation protocol is very specific. Mitochondrial DNA contamination cannot be suspected as neither the Input nor TH2B-associated chromatin was found to be associated with MtDNA. This TH2A–MtDNA association can be explained by the presence of NumtS (Nuclear insertion of Mitochondrial DNA sequences). Reportedly, in mouse, almost 95% of MtDNA is replicated in the nuclear genome, and these are found on almost 15 chromosomes (Malik et al., 2016). It is well established that mitochondrial inheritance is strictly maternal. However, very recently, two independent research labs have published striking evidences for biparental MtDNA inheritance leading to heteroplasmy. Lutz-Bonengel et al. have attributed their finding as well as that of Luo et al. to multiple-copy Mega-NUMTs (Luo et al. 2018; Lutz-Bonengel et al., 2021). Since TH2A is retained in the sperm, there are high chances of it getting transmitted to the zygote along with associated NUMTs. This alliance with NUMTs is perplexing and needs to be further studied.

The replication-independent, non-canonical histone variants TH2A/TH2B are located on chromosome 13 in mice and chromosome 6 in humans (Goldsmith et al., 2005). TH2A (hist1h2aa) lies upstream of TH2B (hist1h2ba) and their transcription is controlled by a common bidirectional promoter (Figure 1A; (Huh et al., 1991)). We observed that a significant number of TH2B peaks were exclusive of TH2A and unique to TH2B. Thus, although they might be sharing a bidirectional promoter between them, they might not be collectively associated in all the nucleosomes and thus will have a distinct epigenetic profile. Recently, it has been shown that phosphorylated TH2A-Thr127 does not preferentially form dimers with TH2B (Hada et al., 2017). TH2B was found to preferentially dimerize with another H2A variant, H2A.L.2 as observed in MNase-sensitive subnucleosomal fraction in elongating and condensing spermatids (Govin et al., 2004; Barral et al., 2017). This dimerization helps in systematic removal of histones for replacement with transition protein and finally protamines. Co-immunoprecipitation of TH2B and H2A.L.2 has also been reported by the same group. In the H2A.L.2. KO, TH2B levels were also altered but H3 and H4 remain unaltered. These studies point to the different chromatin structure of TH2B as compared to TH2A.

### TH2B Is Associated With Spindle Organization and Developmentally Important Genes

Multiple subunit protein complex cohesin is essential for chromosome segregation. It is hydrolyzed *via* separases and the sister chromatin cohesion is disrupted, followed by their pulling apart to the opposite poles *via* spindles. In the double mutant TH2A/TH2B mice, there is an incomplete release of cohesin at interkinesis and accumulation of secondary spermatocytes (Shinagawa et al., 2015). It was also observed that meiosis-specific subunit Rec8 and axial element protein of cohesin complex protein SCP3 remain bound in mutant spermatocytes, thus delaying the entry into meiosis II. Our study found an association between TH2B and spindle assembly. TH2B associations were found along with activating modification H3K4me3 on *Rec8*, *Slx4*, *Hus1b*, and *Sycp2*. This H3K4me3 monovalency may be indicative of active gene expression of these TH2B-associated genes. However, Shinagawa and coworkers have not reported any spindle assembly defects. This could likely be a missed observation. As TH2B promoters in the somatic cells are hypermethylated, its expression is confined exclusively to the germ cells (Choi and Chae, 1991). Thus, this defect in the spindle assembly might be exclusive to germ cells. Many histone variants have overlapping functions. Since in the absence of TH2B its function is taken over by H2B, the effect might not have been pronounced till the interkinesis stage but subsequently the absence of TH2B has manifested the phenotype. It is important to consider that although histone variants may have overlapping functions, the presence and retention of each might be of significance, as nature is not so benevolent to include non-useful protein in such a tightly orchestrated process as spermatogenesis.

The presence of TH2B on developmentally important loci like HOX cluster, sperm lincRNAs, embryonic lethal genes, *Dhh,* and *Sox 30*, as well its overlap with H3K4me3 marks in two-cell embryo, is a significant observation highlighting the importance of sperm-retained TH2B in post-fertilization stages. Since TH2B promotes nucleosomal instability, its presence will lead to early transcription of its associated genes in pre-implantation embryo stages. Previously, there are reports of transmission of paternal histones to the zygotes (Van der Heijden et al., 2008). A paternal TH2B tagged mice will be resourceful to understand the dynamics of sperm TH2B in the pre-implantation stages.

### Mature Sperm-retained TH2B-associated loci overlap with activating modifications H3K4me3/H3K4me2 in sperm and 2-cell embryos.

Knowledge of the post-translational modifications (PTMs) accompanying TH2B marked loci is crucial to unravel the molecular events and significance of TH2B-associated chromatin. With respect to repressive marks H3K27me3, H3K9me3, and H3K36me3, there was almost no overlap with TH2B chromatin. We observed a positive enrichment of H3K4me3 overlapping with the peak sequences. Both H3K4me3and H3K4me2 were enriched (Siklenka et al., 2015; Lambrot et al., 2019; Lismer et al., 2020). We also found enhancer mark H3K27Ac associated with TH2B marked loci. Recent studies have highlighted that sperm H3K4me3 but not H3K27me3 escaped reprogramming in pre-implantation embryonic stages; thus, potential paternal epigenetic inheritance can be mediated by sperm H3K4me3 (Lismer et al., 2020; Oikawa et al., 2020). With this knowledge and the fact that nucleosome is an octamer, we scored our sperm-retained TH2B peaks with the H3K4me3 dataset of the two-cell embryo. It is noteworthy that although slight, we found overlap between the sets (Figure 8). This is indirect evidence of transmission of paternal TH2B to the embryo and needs additional validation.

### Human and Murine Sperm-Retained TH2B and Their Association With Sperm Function-Related Transcripts

It is important to extrapolate the findings in mouse to humans as the former is an experimental model. Also, in the literature as previously stated, there is a range in the histone retention percentage in sperm. For humans, it is 4%–10%, whereas for mice, it varies from ∼1% to 10%. These differences make it important to compare the chromatin occupancy of TH2B between the orthologs. Any conservation may be important from the point of evolution. To highlight the relevance of our findings, we explored the commonality in the dynamics of TH2B between murine and human sperm. A subset of genes important for morphogenesis and embryo development were found common between the two (Figure 9A). Genes such as *Nr4a3*, *Osr1*, and *Zpr1* are important for gastrulation and blastocyst differentiation. N4bp2l2 is crucial for embryo development (Matsuoka et al., 2008). It is expressed at the one-cell stage in mouse and TH2B association with it might be important as it promotes open chromatin, thus aiding early transcription.

Sendler et al. have found sperm RNAs to be associated with H3K4me3 but not H3K27me3 (Sendler et al., 2013). We see a similar trend in the conserved sperm transcripts and TBAGs. Also, the high levels of H3K4me3 marks linked to the sperm transcripts poise these for active transcription for immediate post-fertilization events. These observations could also be indicative of their activity during earlier spermatogenic processes/events. This highlights the importance of TH2B-linked chromatin and also shows conservation across the species and demands further understanding of these orthologous retained chromatin marks. Work has been initiated in that direction.

In summary, our murine TH2A and TH2B ChIP-seq data and *in silico* analysis have attributed a myriad of novel functions to sperm-retained TH2B with respect to embryo development and spermatogenesis. Our data indicate a degree of conservation between the TH2B–DNA linkage across human and mouse. Our observations also convincingly demonstrate that TH2B/TH2A are relatively independent in their genome-wide chromatin associations. The TH2A–MtDNA link observed by us needs to be explored further. *In vivo*, validation of these observations will be crucial to further understand TH2B dynamics.

## Data Availability

The original contributions presented in the study are publicly available. This data can be found here: GEO, accession number: GSE181921.
