# Supplementary figures and images for: Heterogeneity in the Epigenetic Landscape of Murine Testis-Specific Histone Variants TH2A and TH2B Sharing the Same Bi-Directional Promoter
Source: Front Cell Dev Biol. 2021 Dec 6;9:755751. doi: 10.3389/fcell.2021.755751 (PMC8685415; doi:10.3389/fcell.2021.755751)

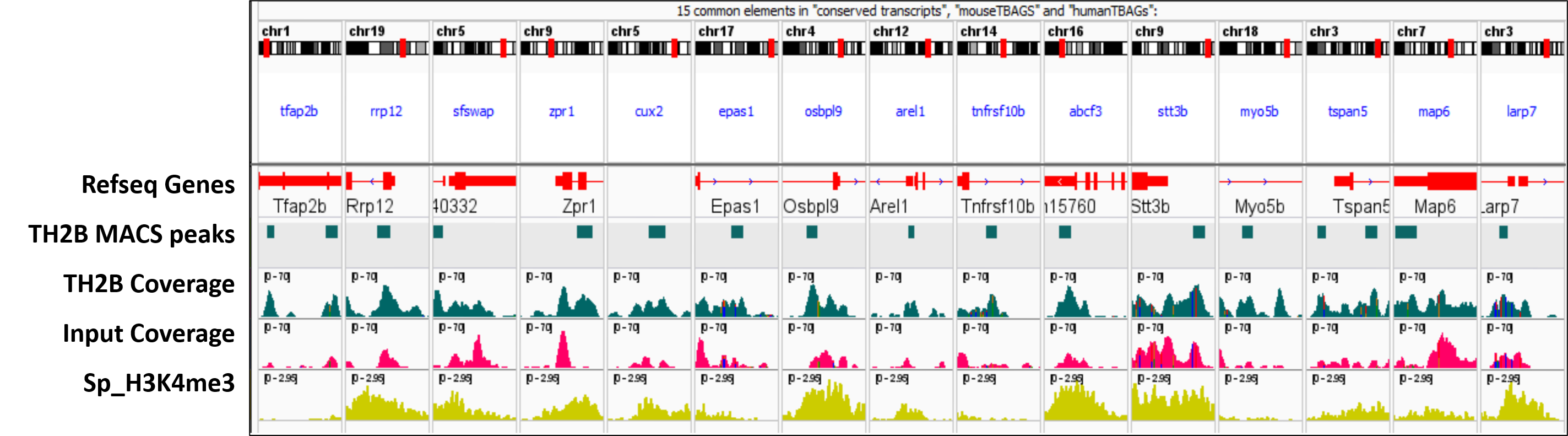

Supplement: Supplementary file 3 [file Image6.TIF]

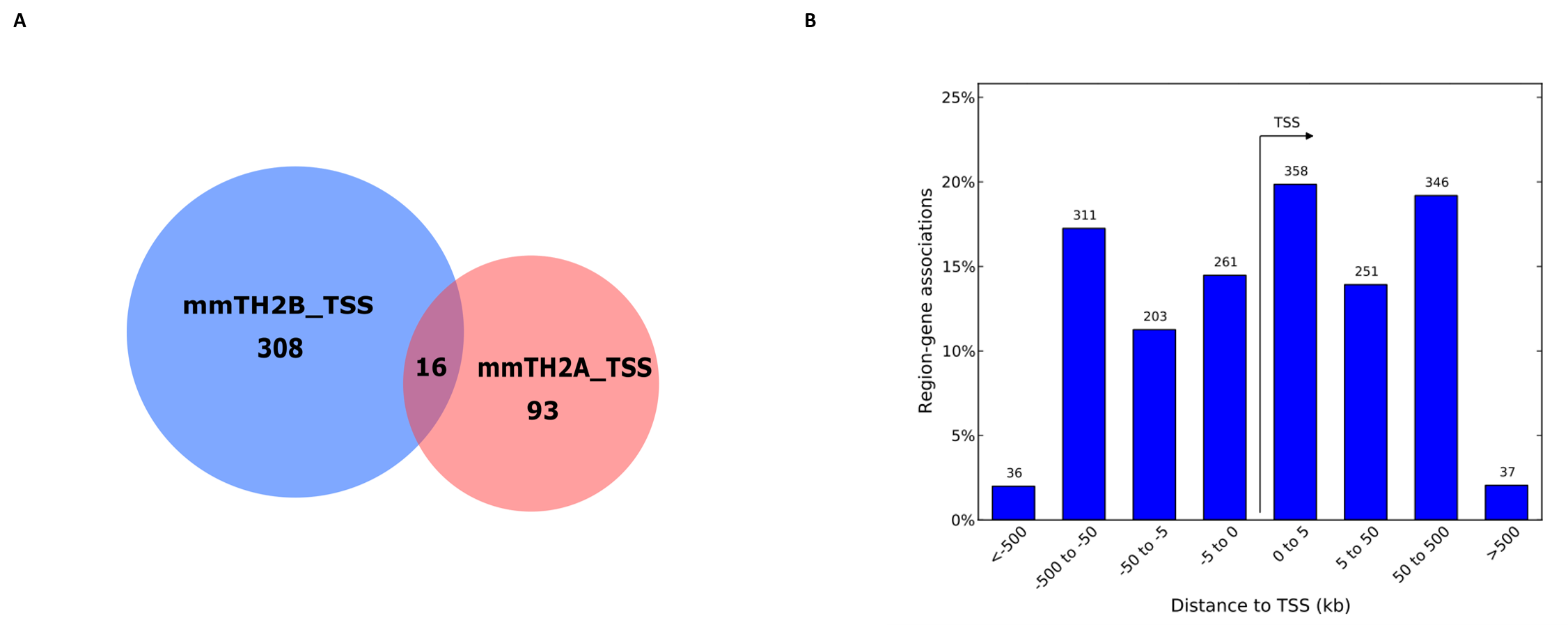

Supplement: Supplementary file 4 [file Image3.TIF]

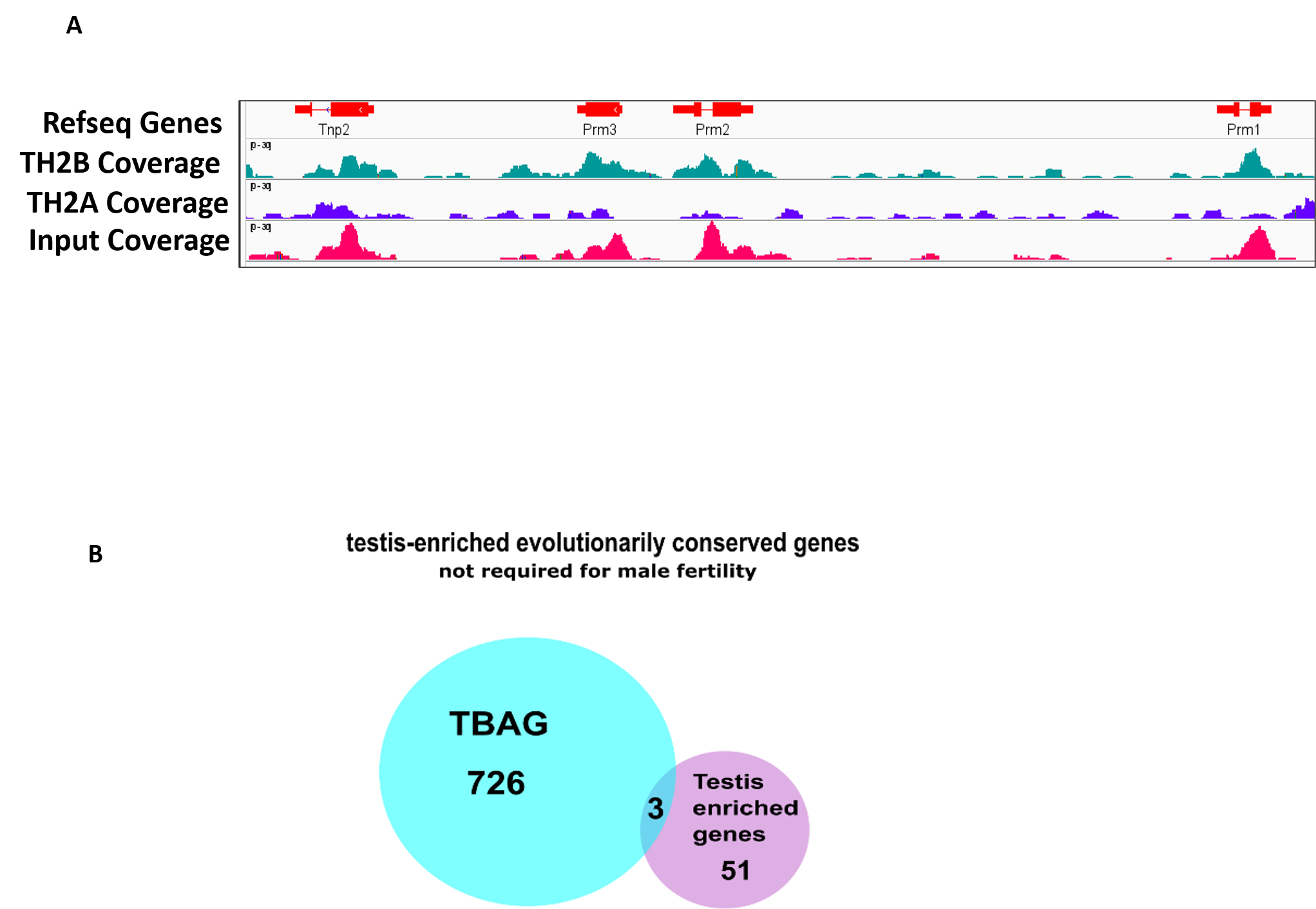

Supplement: Supplementary file 5 [file Image4.TIF]

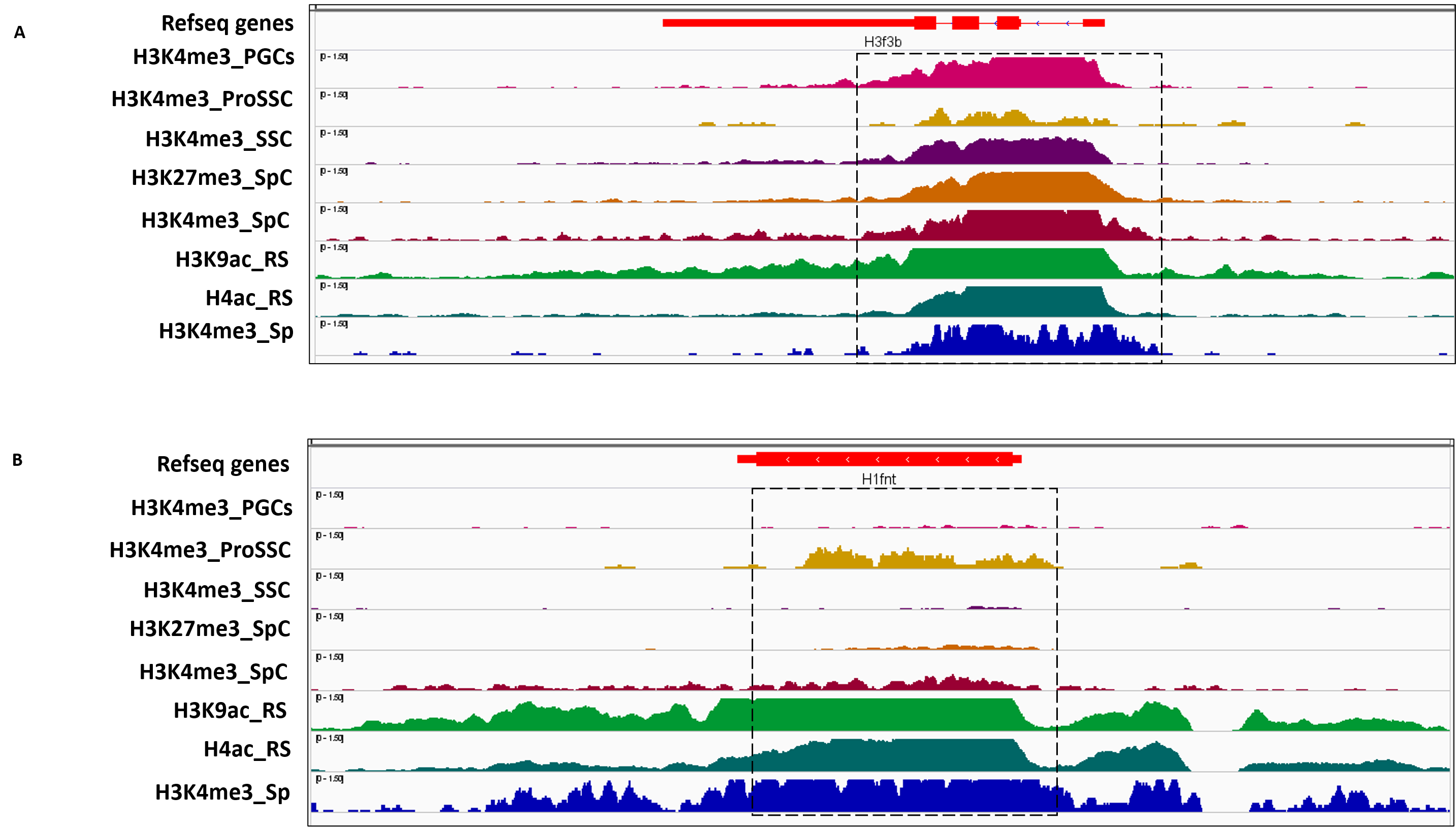

Supplement: Supplementary file 6 [file Image2.TIF]

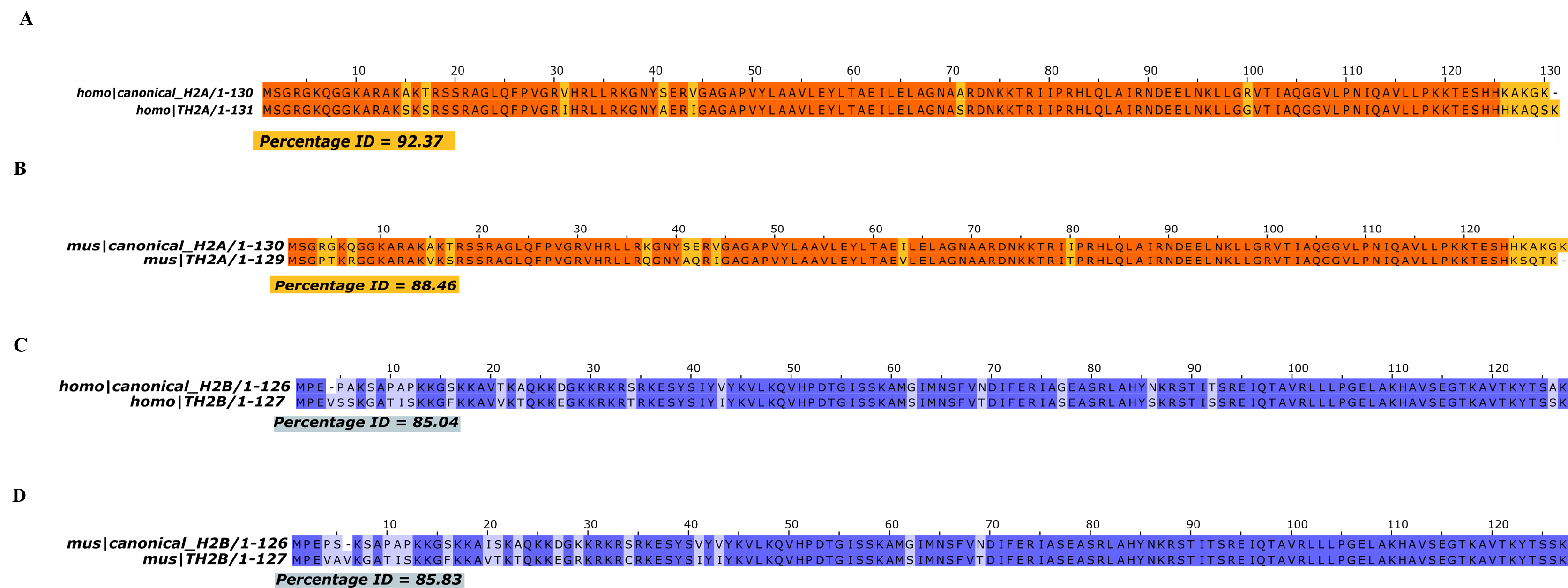

Supplement: Supplementary file 7 [file Image1.TIF]

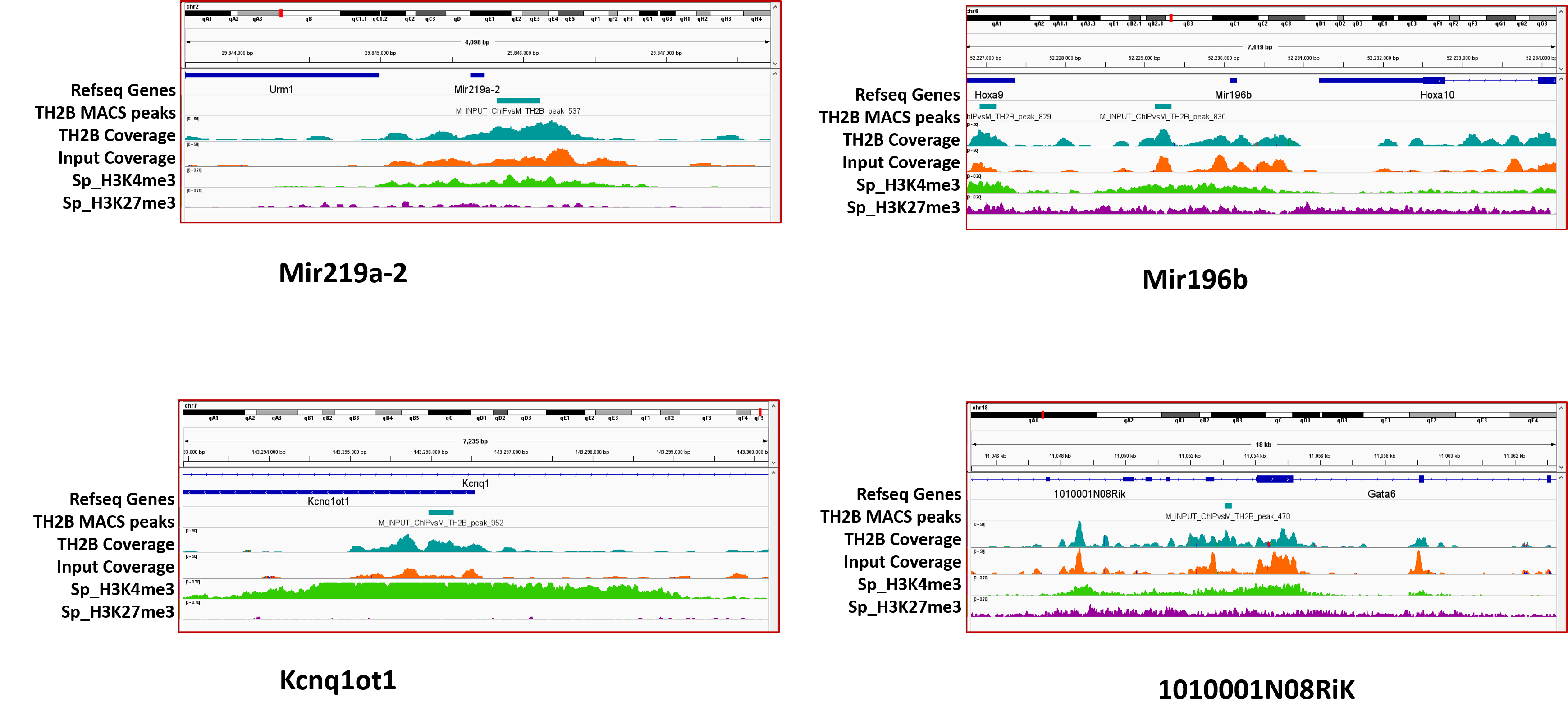

Supplement: Supplementary file 10 [file Image5.TIF]
